# Predicting the Functions of Long Noncoding RNAs Using RNA-Seq Based on Bayesian Network

**DOI:** 10.1155/2015/839590

**Published:** 2015-02-28

**Authors:** Yun Xiao, Yanling Lv, Hongying Zhao, Yonghui Gong, Jing Hu, Feng Li, Jinyuan Xu, Jing Bai, Fulong Yu, Xia Li

**Affiliations:** ^1^College of Bioinformatics Science and Technology, Harbin Medical University, Harbin, Heilongjiang 150086, China; ^2^Key Laboratory of Cardiovascular Medicine Research, Harbin Medical University, Ministry of Education, Harbin, Heilongjiang 150086, China

## Abstract

Long noncoding RNAs (lncRNAs) have been shown to play key roles in various biological processes. However, functions of most lncRNAs are poorly characterized. Here, we represent a framework to predict functions of lncRNAs through construction of a regulatory network between lncRNAs and protein-coding genes. Using RNA-seq data, the transcript profiles of lncRNAs and protein-coding genes are constructed. Using the Bayesian network method, a regulatory network, which implies dependency relations between lncRNAs and protein-coding genes, was built. In combining protein interaction network, highly connected coding genes linked by a given lncRNA were subsequently used to predict functions of the lncRNA through functional enrichment. Application of our method to prostate RNA-seq data showed that 762 lncRNAs in the constructed regulatory network were assigned functions. We found that lncRNAs are involved in diverse biological processes, such as tissue development or embryo development (e.g., nervous system development and mesoderm development). By comparison with functions inferred using the neighboring gene-based method and functions determined using lncRNA knockdown experiments, our method can provide comparable predicted functions of lncRNAs. Overall, our method can be applied to emerging RNA-seq data, which will help researchers identify complex relations between lncRNAs and coding genes and reveal important functions of lncRNAs.

## 1. Introduction

There are only ~1% of human transcripts encoding proteins [1], and a large fraction of transcripts is long noncoding RNAs (lncRNAs), which are an unknown component of mammalian genomes [2]. LncRNAs are spliced, polyadenylated ranging from 200 bp to more than 10 kb [3–5]. They are transcribed from genome regions that are known to lack protein-coding genes, open reading frames, and other properties necessary to be translated into proteins [6, 7]. Recent studies showed that lncRNAs play key roles in many important biological processes, such as the development of vertebrates, cell differentiation, and immune responses, and are related to complex human diseases [2, 8–12]. LncRNAs can have diverse functions in gene regulation, especially in the epigenetic control of chromatin [13–16]. The most famous example is the inactive X chromosome through cis-acting of XIST lncRNA [17]. In addition to cis-regulation, lncRNAs can also act in trans to regulate gene expression [7]. For example, Rinn et al. found that HOTAIR acted in trans to repress HOXD locus transcription [5]. Despite many interesting findings of a few lncRNAs, generalizing these findings to thousands of lncRNAs is difficult. More importantly, the functions of most lncRNAs are largely unknown in comparison to small noncoding RNAs (i.e., microRNAs) [18]. Therefore, predicting functions of lncRNAs remains a greatly substantial challenge.

Currently, there have been some significant efforts applied to identify lncRNAs and explore their functions. Guttman et al. performed loss-of-function experiments on many large intergenic noncoding RNAs expressed in mouse embryonic stem cells and characterized the effects on gene expression [8]. They demonstrated that these noncoding RNAs (ncRNAs) play key roles in the control of embryonic stem cell state. However, these knockdown experiments are time consuming and labor intensive [19]. Some researchers attempt to predict functions of lncRNAs by means of different biological information, such as sequences or genomic positions of lncRNAs. For example, Bellucci et al. utilized lncRNA secondary structure propensities inferred based on sequence information to predict their associated proteins [20] and hence provide a potential way to predict functions of lncRNAs. Wamstad et al. determined GO enrichment for the two nearest neighboring protein-coding genes relative to lncRNAs and found the involvement of lncRNAs in development, morphogenesis, and transcriptional processes [13]. In addition, studies of protein-coding genes have revealed that the relations between mRNAs with similar half-lives have closely related physiological functions, raising the possibility that the half-lives of noncoding RNAs also can be used to identify their functions [21, 22]. Recently, researchers used reannotation microarray expression data to identify lncRNAs and predicted functions of lncRNAs based on coding-noncoding gene coexpression network [23]. However, reannotation microarray strongly depends on the design of the probes [24, 25].

RNA-sequencing (RNA-seq) performs whole transcriptome sequencing and quantifying gene expression with dynamic range, which overcomes the shortcomings of the microarray technology and has already been widely used for studying model organisms and human [9, 26–28]. Cabili et al. defined a reference catalog of more than 8000 human long intergenic noncoding RNAs from RNA-seq data [11], most of which were not previously described. Recent great advances in RNA-seq and computational methods for reconstructing transcriptome offer a wonderful opportunity to annotate and characterize lncRNAs, and a large number of lncRNAs have been discovered using RNA-seq [11, 29, 30]. Therefore, abundant RNA-seq data allow us to comprehensively identify and quantify lncRNAs (also protein-coding genes) and enable us to study the important roles of lncRNAs in various biological processes.

Here, we used RNA-seq of 58 prostate samples to identify lncRNAs and protein-coding genes and construct transcript profiles of lncRNAs and protein-coding genes, respectively. Based on the Bayesian network method, a regulatory network for capturing relations from lncRNAs to protein-coding genes was constructed. Protein-coding gene modules linked with each lncRNA from the regulatory network were identified through mapping to protein interaction network, and its functions were subsequently predicted. A total of 762 lncRNAs were assigned functions. Consistent with previous reports, many lncRNAs are widely involved in the development, cell cycle, metabolism, and other biological processes.

## 2. Materials and Methods

### 2.1. RNA-Seq Data Sets

Fifty-eight prostate samples [31] were detected using RNA-seq, including 42 prostate cancer samples and 16 benign samples. The alignment BAM files that were available at the Gene Expression Omnibus (GEO) database with accession number GSE25183 were directly used for subsequent analysis. We obtained other RNA-seq data of 30 prostate cancer samples (GSE22260) [32]. In addition, the raw RNA-seq data of 32 breast cancer samples was downloaded from GEO with accession number GSE45419 [33]. We also used our previously detected RNA-seq data of brain tissues from 38 psychiatric and normal samples that has been deposited at the Sequence Read Archive (SRA) database (accession number SRP035524) [34].

### 2.2. Protein-Protein Interaction (PPI) Network

Protein-protein interaction network can offer a global view to understand gene functions and various cellular processes. The protein interaction network was obtained from the Human Protein Reference Database (HPRD). We extracted the maximum component of the protein interaction network, which contained 36900 interactions and 9219 genes.

### 2.3. Construction of Transcript Profiles of lncRNAs and Protein-Coding Genes

**Figure 1:**
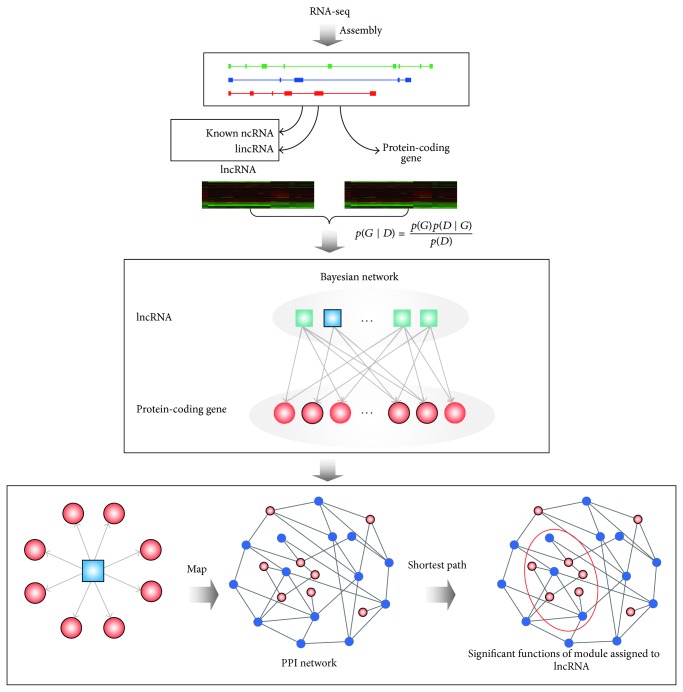
The workflow of functional prediction of lncRNAs. We identified lncRNAs and protein-coding genes from RNA-seq data of 58 prostate cancer samples and created transcript profiles of lncRNAs and protein-coding genes for construction of the regulatory network between lncRNAs and protein-coding genes based on the Bayesian network method. To predict functions of individual lncRNA in the regulatory network, we mapped its linked protein-coding genes onto human PPI network and mined highly connected modules, which was subsequently used to predict functions by functional enrichment (*P* value < 0.05).

The sequenced reads were mapped to human reference genomic utilizing TopHat [35]. Then, we used Cufflinks [36] to assemble exonic and splice-junction readings into transcripts using their alignment results from TopHat and estimated transcript abundances in fragments per kilobase of exon per million fragments mapped (FPKM) by parsimonious allocations of readings to the transcripts. Subsequently, we used known annotation information from UCSC and Ensembl database to identify ncRNAs, lincRNAs, and protein-coding genes. We combined known ncRNAs and lincRNAs to obtain more comprehensive annotation of lncRNAs (Figure 1).

The assembly results were classified based on the following.

- Genes have at least 90% overlap with known annotation of pseudogenes, which are considered as pseudogenes in our study.
- Genes that do not pass the above step are then compared with the annotation of known noncoding RNAs and those which have at least 90% overlap with known noncoding RNAs are retained as ncRNAs.
- Genes that do not pass the above steps and have at least 90% overlap with long intergenic noncoding gene are retained as lincRNAs.
- The set of genes following the above steps was then compared with the annotation set of known protein-coding genes, if one gene has 60% overlap with known protein-coding genes considering coding gene.
- The remaining genes are unannotated and are thus excluded from our study.

In order to explore potential lncRNA-gene relations, lncRNAs and protein-coding genes were considered for further analysis only if they were expressed in at least 50 samples. Finally, we reserved these lncRNAs longer than 200 bp and constructed transcript profiles of lncRNAs and protein-coding genes. FPKM of lncRNAs and protein-coding genes were set to 0 when they were not present in some samples.

### 2.4. Construction of Bayesian Network

**Figure 2:**
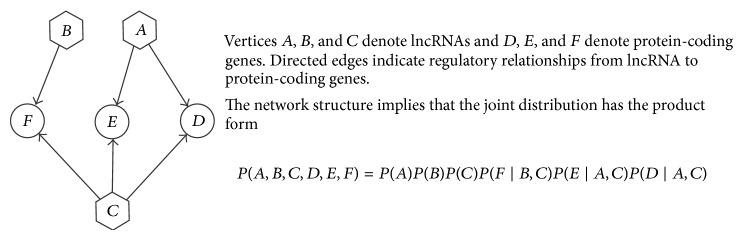
An example of a simple Bayesian network structure.

In this study, we used Bayesian network to reveal regulatory relationships between lncRNAs and protein-coding genes, which has been widely used for discovering gene regulatory networks [37–41]. Bayesian network represents a joint probability distribution as a directed acyclic graph. It consists of two components. The first component, *G*, is a directed acyclic graph (DAG) whose vertices represent the random variables *u*_1_, *u*_2_,…, *u*_*N*_ and whose edges correspond to dependencies between variables. The second component describes a conditional distribution for each variable which is only dependent on its parent vertices. These two components specify a unique distribution on *u*_1_, *u*_2_,…, *u*_*N*_. This joint distribution can be decomposed into a product of conditional probabilities based on the graphical structure:

(1) $$\begin{array}{c} p\left(u_{1},u_{2},\ldots ,u_{N}\right)=\overset{N}{\underset{i=1}{\prod }}p\left(u_{i}\;|\;{\text{Pa}}^{G}\left(u_{i}\right)\right), \end{array}$$

where Pa^*G*^(*u*_*i*_) is the set of parents of *u*_*i*_ in *G*.  Figure 2 shows an example of a Bayesian network *G* and the joint probability distribution it implies.

**Figure 3:**
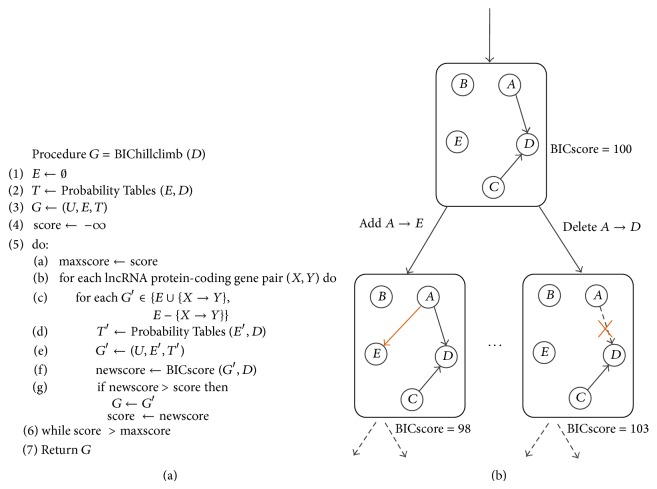
Pseudocode (a) and illustration (b) of a Bayesian network structure hill-climbing search procedure. *E*, *G*, *D*,  and  *U*, respectively, are edge sets, Bayesian graph, the training data, and a subset of vertices in Pseudocode (a).

To construct the regulatory network, we discretized the expression levels of each lncRNA and protein-coding gene from continuous values into two categories (high expression and low expression) by Hartemink's pairwise mutual information method [42]. Then these probability distributions in* formula*  (1) can be computed by counting the frequencies of different combinations. In the process of Bayesian network structure learning, the most likely graph *G* for a given data set *D* can be inferred by searching for the optimal graph based on a Bayesian scoring metric. As both structure and parameters of the Bayesian network are typically unknown [43], we thus employed the commonly used approximate Bayesian scoring metric, Bayesian information criteria (BIC) [44, 45]. The BIC scoring function can be defined as follows:

(2) $$\begin{array}{c} \text{BICscore}\left(G,D\right)=\log L\left(u_{1},u_{2},\ldots ,u_{N}\right)-\frac{d}{2}\log n, \end{array}$$

where *L*(*u*_1_, *u*_2_,…, *u*_*N*_) is the likelihood of the data *D* according to estimated parameters and structure *G*, *n* is the sample size of the data set, and d is the number of parameters. Finally, the graph space was explored using the greedy hill-climbing algorithm with random restarts to get the most likely graph *G* for lncRNAs and protein-coding genes. Pseudocode and illustration for hill-climbing algorithm are shown in Figure 3.

The construction of Bayesian network was generated using *R* package bnlearn [45].

### 2.5. Prediction of the Functions of lncRNAs by Network Modules

In general, genes with high interconnections tend to have more similar functions [46]. Therefore, predicting the function of lncRNAs based on their directly linked protein-coding genes together can benefit from network modular strategies [47, 48] because lncRNAs may be involved in multiple biological processes.

We integrate the protein-protein interaction (PPI) network and then predict the functions of lncRNAs using network modules derived from the PPI network. For each lncRNA in the regulatory network constructed based on the Bayesian network method, its directly linked protein-coding genes were mapped onto the human PPI network. Of these genes in the PPI network, we computed the shortest path lengths between any two genes and then used the dynamic cutting tree [49] to mine gene modules. The significant functions enriched by each module (*P* value < 0.05) were identified. These functions were regarded to be associated with the lncRNA.

## 3. Result

### 3.1. Construction of Transcript Profiles of lncRNAs and Protein-Coding Genes Using RNA-Seq

RNA-seq provides an accuracy and dynamic characterization of the whole cell transcriptome, including different types of RNAs, such as mRNAs and lncRNAs. A total of 58 prostate samples were detected using RNA-seq [31]. The size of 250–300 bp polyA-RNA fragments was selected to construct libraries, which were sequenced using single-end and paired-end on an Illumina Genome Analyzer I and Genome Analyzer II flow cell. In total, approximately 300 million readings were mapped to human reference genome hg18 using TopHat with default parameters. The alignment BAM files that were available at the Gene Expression Omnibus (GEO) database with accession number GSE25183 were directly used for subsequent analysis (Figure 1).

To construct transcript profiles of lncRNAs and protein-coding genes, these mapped readings were assembled into transcripts using Cufflinks [36] with default parameters (a maximum intronic length of 300 kb; minor isoforms with abundance less than 10% of the major isoform). For all 58 samples, 1.69 million transcripts were generated from Cufflinks corresponding to 59404 genes (Supplemental Table S1 available online at http://dx.doi.org/10.1155/2015/839590). We annotated these millions of magnitude of transcripts generated by Cufflinks based on known genomic annotation information from different databases (e.g., UCSC and Ensembl). These genomic annotations are composed of the following: (1) 18921 protein-coding genes from UCSC database coding gene track; (2) 37584 ncRNAs from type of noncoding transcripts in Ensembl database; (3) 8669 pseudogenes obtained through combination of the annotation of pseudogenes in UCSC and Ensembl database; and (4) 21552 lincRNAs from the UCSC lincRNA track.

**Figure 4:**
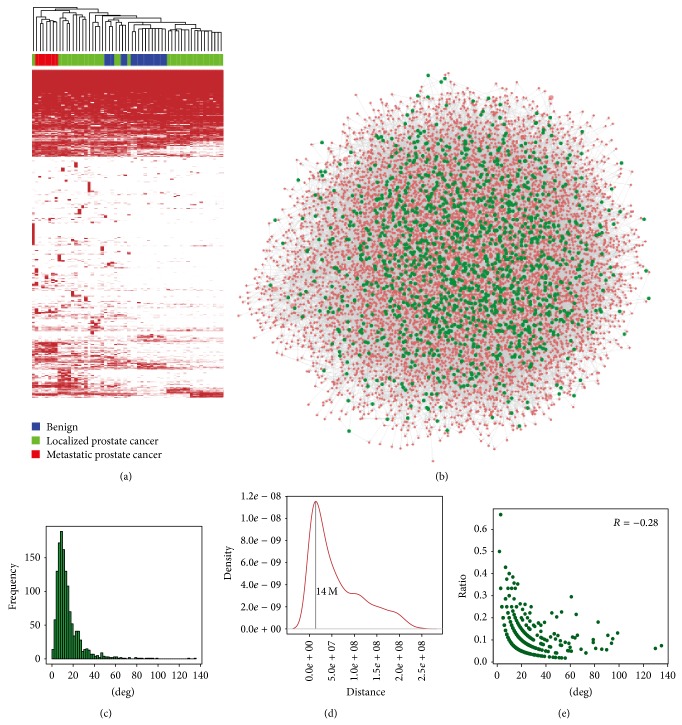
(a) A binary map showing the presence (red) and absence (white) of lncRNAs in 58 samples can distinguish prostate cancer samples from benign samples and differentiate localized prostate cancer samples from metastatic samples. (b) The regulatory network between lncRNAs and protein-coding genes was constructed using RNA-seq data of 58 prostate samples based on the Bayesian network method. Red nodes represent protein-coding genes, and green nodes represent lncRNAs. (c) Distribution of degree of lncRNAs in the regulatory network. (d) The distance between lncRNAs and their linked protein-coding genes. (e) The ratio of protein-coding genes located on the same chromosome with their linking lncRNAs.

Based on known annotation information of protein-coding genes, ncRNAs, pseudogenes, and lincRNAs, transcripts in each sample were classified into sets of protein-coding genes, lincRNAs, known ncRNAs, pseudogenes, and unannotated transcripts. NcRNAs with length greater than 200 bp and lincRNAs were both regarded as lncRNAs. We identified 7843 lncRNAs (6267 from the ncRNA annotation and 1576 from the lincRNA annotation) and 15305 protein-coding genes from the 58 prostate samples according to four filtering rules (details in the method section). We found large variance of lncRNAs across all samples; that is, more than 60.7% of lncRNAs (4763 of 7843) occur at just a few samples (≤10) and only 1355 lncRNAs (17.3%) are present at more than 50 samples. Interestingly, the binary map showing the presence and absence of lncRNAs was able to successfully distinguish normal, metastatic, and localized prostate samples (Figure 4(a)), suggesting the existence of phenotype-specific lncRNAs. In fact, we observed that some lncRNAs trended to be expressed only in metastatic prostate cancer samples and some are expressed only in localized prostate cancer samples. To characterize potential lncRNA-gene relations, lncRNAs and protein-coding genes were obtained only if they were expressed in at least 50 samples. Finally, we obtained 1355 lncRNAs and 8644 protein-coding genes, which were subsequently used to construct a lncRNA-gene regulatory network.

### 3.2. Construction of lncRNA-Gene Regulatory Network Based on Bayesian Network Method

Network analysis offers an efficient method of functional annotation of various biological molecules [50, 51]. Using transcript profiles of lncRNAs and protein-coding genes derived from RNA-seq data, we constructed a lncRNA-gene regulatory network based on the Bayesian network method. We discretized transcript profiles of lncRNAs and protein-coding genes using Hartemink's pairwise mutual information method. Then, using a hill-climbing greedy search on the space of the directed acyclic graph, the optimal network matching transcript profiles of lncRNAs and protein-coding genes were identified. There are 20957 edges referring to 9999 nodes composed of 1355 lncRNAs and 8644 protein-coding genes in the regulatory network containing the dependency relationships between lncRNAs protein-coding genes (Figure 4(b)). We analyzed the distribution of lncRNA degree in the regulatory network (Figure 4(c)), finding that the degree of most lncRNAs is small (mean degree of all lncRNAs is 15.46); only several lncRNAs have large degree (maximum degree of lncRNA is 135). Also, we observed that one lncRNA can connect many protein-coding genes, and one protein-coding gene can also be connected by several lncRNAs.

Previous studies have suggested that lncRNAs can act in cis to activate or silence transcription of genes, such as cis-acting of Xist [17]. However, there is mounting evidence showing that lncRNAs can act in trans, such as HOTAIR silencing HOXD locus [5]. Therefore, we sought to analyze whether lncRNAs tend to affect protein-coding genes in cis or in trans. For each lncRNA in the regulatory network, we extracted its linked protein-coding genes and examined how frequent the linked protein-coding genes located at the same chromosome as the lncRNA. We found only a small part of protein-coding genes located at the same chromosomes as their associated lncRNAs. We further found that lncRNAs affect protein-coding genes with distance around 10–20 Mb with a peak of 14 M (Figure 4(d)), which is more than 100 kb used by analysis of lncRNAs based on neighboring genes [8]. Interestingly, only 15 (1.11%) of 1355 lncRNAs linked with protein-coding genes within 100 kb were identified, suggesting that lncRNAs may act on broader regions in cis, although most genes are probably affected by lncRNAs in trans. Additionally, we found that lncRNAs with larger degrees connect less frequently with protein-coding genes on the same chromosome (Figure 4(e)), suggesting that some hub lncRNAs may function dependently on their effects in trans.

Considering the existence of disease and normal samples in these 58 prostate samples, we used the significance analysis of microarrays (SAM) method [52] to identify differentially expressed lncRNAs and protein-coding genes (false discovery rate, FDR ≤ 0.05). Of 1355 lncRNAs, 510 (37.6%) showed differential expression with 351 upregulated and 159 downregulated lncRNAs. Of 8644 protein-coding genes, 3821 (44.2%) showed differential expression with 2295 upregulated and 1526 downregulated coding genes. Among 20957 edges in the regulatory network, there are 2167 edges which show consistent upregulation, 885 edges with consistent downregulation, 838 edges with upregulated lncRNAs and downregulated genes, and 983 edges with downregulated lncRNAs and upregulated coding genes (Supplemental Figure S1A). As expected, upregulated lncRNAs tend to connect with upregulated protein-coding genes (Wilcoxon's rank sum test, *P* value < 2.2*e* − 16, Supplemental Figure S1B), but not vice versa.

**Figure 5:**
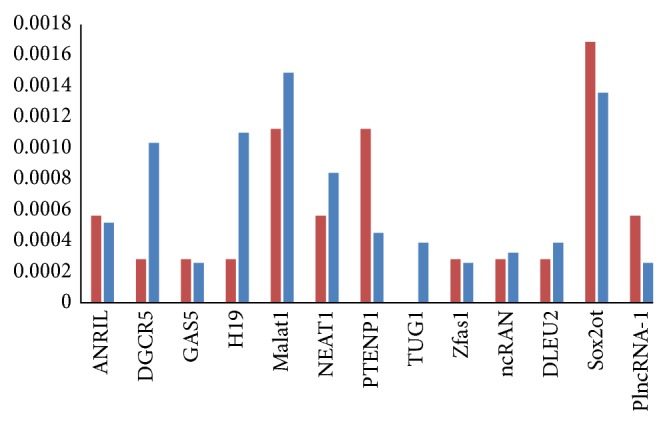
There are 12 disease-associated lncRNAs in the regulatory network. Red bar denotes the proportion of disease genes linked by the disease-associated lncRNAs in all disease genes derived from OMIM. Blue bar denotes the proportion of nondisease genes linked by the disease-associated lncRNAs in the protein-coding genes derived from UCSC.

In addition, recent studies showed that dysregulated lncRNAs contribute to many human diseases [31, 53, 54]. A possible hypothesis is that dysfunction lncRNAs may destroy some known disease genes, which in turn induce the development of disease. Therefore, we assessed whether disease-related lncRNAs are connected with known disease genes in the regulatory network. Through manual literature searching, we found 12 disease-associated lncRNAs (including ANRIL, DGCR5, GAS5, H19, Malat1, NEAT1, TUG1, Zfas1, ncRAN, DLEU2, Sox2ot, PTENP1, and PlncRNA-1) [53] in the network and 7655 disease genes derived from OMIM. We did not find obvious difference between the disease and nondisease genes linked by disease-associated lncRNAs (Figure 5). By comparing the mean degrees of nondisease-associated lncRNAs and disease-associated lncRNAs, we found that the mean degree of disease-associated lncRNAs (mean degree 12.31) is lower than nondisease-associated lncRNAs (mean degree 15.5).

### 3.3. Predicting Functions of lncRNAs Based on Network Modules

To predict the functions of lncRNAs, we applied a module-based method that has been extensively used to predict functions of gene sets through integrating our inferred lncRNA-gene regulatory network and protein-protein interactions. For each lncRNA in the regulatory network, their linked protein-coding genes were mapped onto the human PPI network. On the basis of the PPI network, we computed the shortest path lengths between any two genes to mine modules. The significant functions enriched by each module (*P* value < 0.05) were regarded to be associated with the lncRNA and assigned to the lncRNA.

Of the 1355 lncRNAs in the network, 762 were assigned with enriched functions. The other lncRNAs were not because their linked protein-coding genes either cannot form modules in the PPI network or are not significantly involved in any biological processes. Consistent with previous studies, many of these lncRNAs were found to be associated with development, metabolism, and some fundamental cellular functions (e.g., cell cycle, signal transduction, and transcription) [9, 23]. Using our method can assign functions to known prostate-associated lncRNAs. For example, one lncRNA named PlncRNA-1 has been demonstrated to be related to prostate cancer. A recent study suggests that silencing of PlncRNA-1 significantly reduced cell proliferation and induced apoptosis [54]. Consistently, through our prediction method, we annotated PlncRNA-1 with functions of cell cycle. In the network, we also found that PlncRNA-1 can affect POFUT1 gene involved in Notch signaling pathway, which is required for normal prostatic epithelial cell proliferation and differentiation [55].

**Figure 6:**
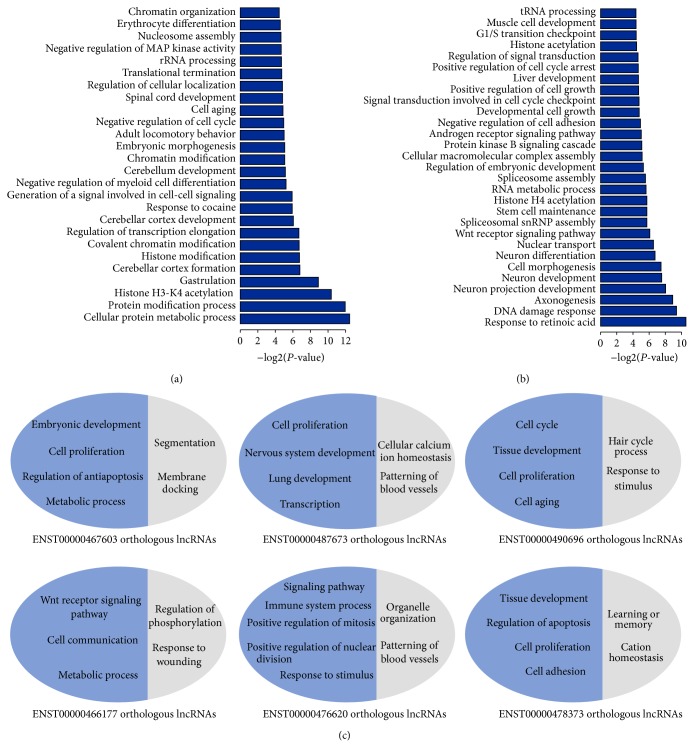
(a) Functional enrichment of Malat1 lncRNA. (b) Functional enrichment of NEAT1 lncRNA. (c) Functions predicted by our method overlapping with those determined in the lncRNA knockdown experiments. Blue color represents overlapping functions, and gray color represents functions only predicted by knockdown experiments.

By using relationships between lncRNAs and protein-coding genes from the regulatory network, we can also annotate poorly characterized lncRNAs with novel functions. For example, Malat1 lncRNA linked 27 protein-coding genes in the network, which form two modules in the PPI network. Genes in the two modules were significantly involved in biological processes, including cerebellar cortex formation, nucleosome assembly, cell cycle, transcription elongation, and cell-cell signaling (Figure 6(a)). Consistently, previous studies have suggested that Malat1 lncRNA is a component of nuclear bodies and may be associated with the cerebellum of human alcoholics, depletion of which resulted in aberrant mitosis increased cell death [56–59]. The lncRNA Malat1 is dispensable for mouse development [60], which consists of our prediction functions of embryonic morphogenesis. In addition, we found several novel functions of Malat1 lncRNA, such as cell aging, histone modification, and metabolic process. Another lncRNA named NEAT1 linked with 15 protein-coding genes in the network forms one module in PPI network form, which was significantly involved in functions related to neuron projection development, cell differentiation, DNA damage response, cell cycle, Wnt receptor signaling pathway, and nuclear transport (Figure 6(b)). Previous studies have suggested that NEAT1 lncRNA has an important structural role in the nuclear paraspeckles [61] and it plays important roles in Huntington's disease by disrupting neuron differentiation [62], which coincides with our predicted functions. A recent study found that NEAT1 might be a general feature of differentiation [62–64], supporting our predicted function of cell differentiation. Interestingly, novel functions, such as cell cycle and DNA damage response, suggest that NEAT1 lncRNA may be associated with the parthenogenesis of cancer. Further experiments could help to elucidate its roles in cancer.

Furthermore, we analyzed the affected biological processes of lncRNAs through their nearest neighboring genes. We identified the nearest neighboring protein-coding genes of these 1355 lncRNAs in the network and completed the functional enrichment using all of these protein-coding genes. The set of neighboring genes is significantly involved in 263 GO terms (*P* value < 0.05), such as metabolic process, development, and cell cycle, consistent with previous reports [11, 65]. Of these enriched GO terms, 89.3% were also found using our method. For each lncRNA in the network, we examined the overlaps between GO terms associated with its nearest protein-coding gene and ones predicted using our method. We found that 132 of 762 lncRNAs have at least one shared GO term, but the overlapping GO terms only occupy a small proportion of terms predicted using our method.

Through knockdown experiments of lncRNAs and subsequent microarray-based expression profiling, a recent study systematically analyzed functions of 147 lncRNAs in mice [8]. To further evaluate the performance of our approach, we searched for orthologous lncRNAs with knockdown data in mice and evaluated the overlapping of functions identified between our approach and knockdown-based experiments. Based on sequence alignment, 48 orthologous lncRNAs were obtained. For each lncRNA, its knockdown expression data were used to determine the affected genes by differential expression analysis (fold change > 2 and *t*-test with FDR < 0.05). We then performed GO enrichment analysis based on the affected genes for the determination of its functions. Among these 48 lncRNAs, we found that 38 lncRNAs show overlapping of functions identified between our approach and knockdown-based experiments; 4 were not involved in any functions based on knockdown expression data, and 5 were not based on our approach. Also, we found that our approach can capture many important functions (e.g., development, cell proliferation, and cell differentiation) that were also confirmed by knockdown-based experiments (Figure 6(c), Supplemental Figure S2). For example, by analyzing the knockdown expression data of ENST00000467603 orthologous lncRNA in mice, we found the orthologous lncRNA involved in embryonic development, cell proliferation, and cell death processes (Figure 6(c)) that were also predicted using our approach. In particular, we found that the lncRNA connects with the IRF6 gene in the regulatory network, which encodes a member of the interferon regulatory transcription factor (IRF) family involved in the development process [66, 67] and regulation of cell proliferation [68]. Consistently, the knockdown of its orthologous lncRNA in mice significantly affected the expression level of the ortholog gene of IRF6. By analyzing the knockdown expression data of another lncRNA named ENST00000487673 orthologous lncRNA in mice, we found the orthologous lncRNA involved in cell proliferation, lung development, and tissue morphogenesis processes, which were also predicted using our method. Moreover, in the regulatory network, the ENST00000487673 lncRNA was found to link with SLC39A6 gene, an essential cofactor for hundreds of enzymes, encoding one member of the SLC39 family and involved in differentiation and development [69, 70]. Notably, knockdown of its orthologous lncRNA in mice significantly affected the expression level of SLC39A8 gene, whose orthologous gene in human together with SLC39A6 belong to the SLC39 family [69].

### 3.4. Application of the Method to Other RNA-Seq Data

**Figure 7:**
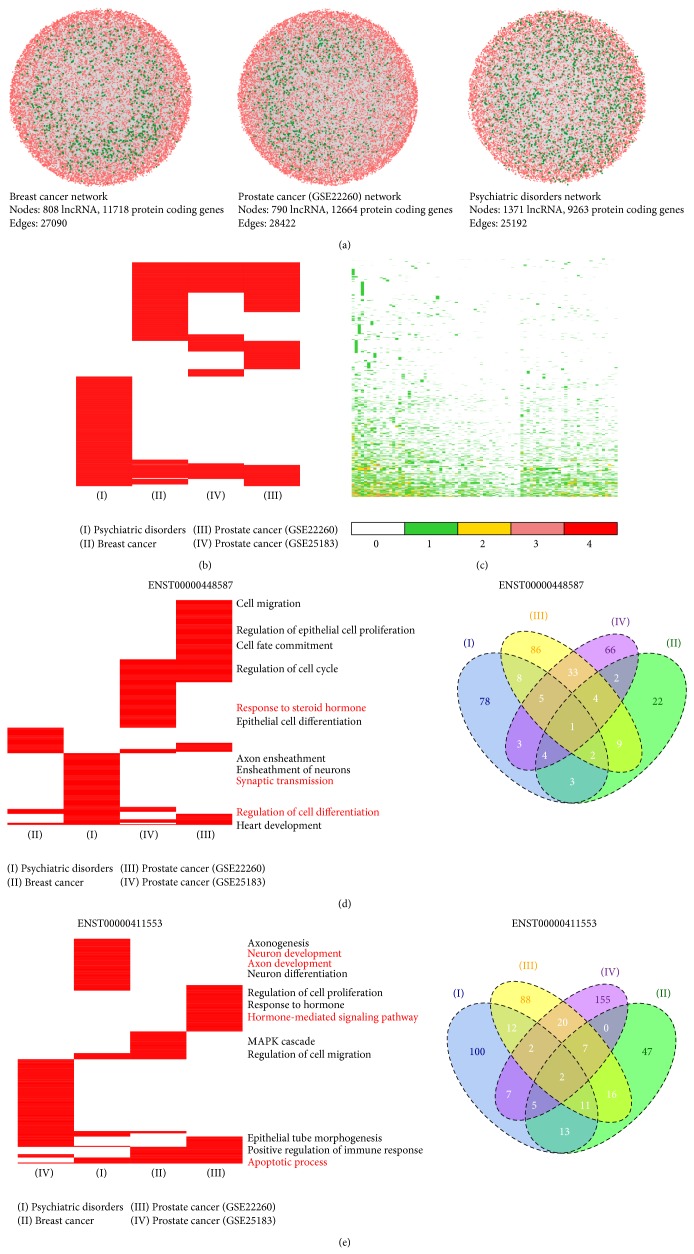
(a) Regulatory networks between lncRNAs and protein-coding genes were, respectively, constructed using RNA-seq data of breast cancer, prostate cancer, and psychiatric disorders based on the Bayesian network method. Red nodes represent protein-coding genes, green nodes represent lncRNAs, and edges represent regulatory relationships. (b) Heatmap representing the GO terms of lncRNAs in four data sets. For each lncRNA, detected GO terms are indicated in red. I denotes psychiatric disorders, II denotes breast cancer, III denotes prostate cancer (GSE22260), and IV denotes prostate cancer (GSE25183). (c) Heatmap shows the shared functions of lncRNAs in four data sets (columns). Color key represents the number of data sets sharing the same GO terms for each lncRNA (rows). (d) Functional heatmap (left panel) representing the GO terms (rows) of ENST00000448587 in four data sets (columns). Detected GO terms in each data set are indicated in red. Venn diagram (right panel) showing the number of overlapped GO terms of ENST00000448587 in four data sets. (e) Functional heatmap (left panel) representing the GO terms (rows) of ENST00000411553 in four data sets (columns). Detected GO terms in each data set are indicated in red. Venn diagram (right panel) showing the number of overlapped GO terms of ENST00000411553 in four data sets.

We applied our method to three other RNA-seq data sets including breast cancer, prostate cancer, and psychiatric disorders and obtained their corresponding regulatory networks. There were 27090 edges referring to 12526 nodes composed of 808 lncRNAs and 11718 protein-coding genes in the regulatory network of breast cancer, 28422 edges referring to 13454 nodes composed of 790 lncRNAs and 12664 protein-coding genes in prostate cancer, and 25192 edges referring to 10634 nodes composed of 1371 lncRNAs and 9263 protein-coding genes in psychiatric disorders (Figure 7(a)). For each regulatory network, we predicted the functions of lncRNAs by using network modules derived from the PPI network. A total of 668, 648, and 717 lncRNAs were assigned with enriched functions in breast cancer, prostate cancer, and psychiatric disorders, respectively. We found that some lncRNAs commonly appeared in these four RNA-seq data sets (Figure 7(b)), and some lncRNAs were present in only one RNA-seq data set, in line with high tissue specificity of lncRNAs [11]. As expected, the two RNA-seq data sets of prostate cancer shared more lncRNAs than others (Figure 7(b)). To compare the functions of lncRNAs among these four RNA-seq data sets, we obtained 85 common lncRNAs. We found that lncRNAs can be involved in the same functions among these four RNA-seq data sets and, notably, they can also be enriched in some tissue-related functions (Figure 7(c)). For example, lncRNA ENST00000448587 (known as TINCR) was found to be enriched in heart development among these four data sets (Figure 7(d)). In particular, we found that the lncRNA was also involved in regulation of cell differentiation in three data sets including breast cancer, prostate cancer, and psychiatric disorders, which was consistent with a previous study that TINCR can control tissue differentiation [71]. Moreover, we found that the lncRNA was enriched in tissue-related functions, such as synaptic transmission and axon ensheathment in psychiatric disorders and response to steroid hormone in prostate cancer. In these four RNA-seq data sets, lncRNA ENST00000411553 was enriched in apoptotic process, suggesting its importance in the development of diseases. We also found that ENST00000411553 was involved in tissue-related functions such as neuron differentiation, axonogenesis, and neuron development in psychiatric disorders, hormone-mediated signaling pathway in prostate cancer, and regulation of cell migration in breast cancer (Figure 7(e)).

## 4. Discussion

Although human genome encodes thousands of lncRNAs, only a few lncRNAs have been functionally characterized, and most functions of lncRNAs remain unknown. Here, we proposed an integrative framework to systematic prediction of lncRNA function. Using combination of a large number of RNA-seq data sets, we constructed a lncRNA-gene regulatory network based on the Bayesian network method and then predicted lncRNA functions using a module-based strategy by integrating protein interaction network. Our results show that lncRNAs are involved in diverse biological processes, such as development, metabolism, and differentiation, consistent with many previous studies.

Recently, some researchers used reannotation microarray expression data to predict functions of lncRNAs [23, 72]. However, microarray is greatly dependent on designed probes and hence cannot comprehensively characterize dynamic and relatively low expression of lncRNAs [24, 25]. Also, lncRNAs have strong tissue specificity [9, 11], and many lncRNAs are not identified at present [29]. RNA-seq has the ability to capture the expression levels of genome-wide transcripts, including ones with extremely low expression levels, which thus provides a more precise measurement of levels of transcripts with great dynamic range in comparison to microarray [27, 50]. Expression levels of lncRNAs detected using RNA-seq cannot be reproduced using microarray because of their low correlation [73]. More importantly, RNA-seq data can be used to identify known and novel lncRNAs [11, 29] and quantify their transcript abundance [27] in a specific condition [31]. Therefore, our approach—utilization of RNA-seq data—has the ability to characterize condition-specific lncRNAs and mRNAs, which can further help to systematically depict their potential relations.

With the Bayesian network method as our basis, we constructed a lncRNA-gene regulatory network using transcript profiles of lncRNAs and protein-coding genes generated from RNA-seq data. The Bayesian network allows us to discover causal relations between lncRNAs and genes by capturing properties of conditional independence between variables [37]. It also allows us to handle noise and focus on dependency relationships with strong signals in observed data. It has been widely used for building a variety of regulatory networks [37, 40, 74].

Subsequently, we applied a module-based strategy through combination of the lncRNA-gene regulatory network and protein interaction network. Such modular method has been widely used in prediction of molecular functions because of the prevalence of modular organization of biological networks [51]. LncRNAs may also exert specific functions by regulating function-related genes or by regulating key genes, which in turn affect downstream function-related genes. Using the module-based method, genes connected by a given lncRNA in the regulatory network were divided into coherent groups of genes that show tight connections in protein interaction network. Thus, our method can effectively predict lncRNA functions by considering not only causal relations between lncRNAs and protein-coding genes but also functional associations between genes in protein interaction network.

Furthermore, taking into account cis-acting of lncRNAs, several studies used their neighboring protein-coding genes to annotate the function of lncRNAs. However, it is difficult to determine the genomic range of cis-acting for lncRNAs. That is, there is absence of a unified criterion for the establishment of neighboring protein-coding genes of lncRNAs. Previous studies used different distances to search for their neighboring genes, such as 10 kb [28, 75] and 300 kb [8], and also used one (or two) nearest neighboring protein-coding gene(s) to describe the function of lncRNAs [11, 13]. Moreover, it is difficult to predict functions of individual lncRNAs dependent on one or a few neighboring genes. More importantly, a recent loss-of-function experiment has been used to investigate the effects of lncRNAs on protein-coding gene expression [8]. They found that only 2 of 147 lincRNAs function in cis and most lincRNAs affect gene expression in trans. The trans-acting of lncRNAs will hinder the function characterization of lncRNAs based on neighboring protein-coding genes. In comparison with neighboring gene-based function prediction of lncRNAs, we found a part of lncRNAs with functions predicted using our method overlapping with those predicted by their neighboring genes. Furthermore, we found that our method can also capture most functions of orthologous lncRNAs in mouse determined by lncRNA knockdown experiments. Obviously, our method is not restricted to potential cis-acting of lncRNAs and thus can be used to explore more extensive functions for lncRNAs.

In addition, in the regulatory network, 8644 (45.7%) of 18921 protein-coding genes are linked by lncRNAs, suggesting broad effects of lncRNAs on protein-coding genes and therefore supporting their important roles in biology [8]. Interestingly, we found 10 of the 12 disease-associated lncRNAs linked with at least one disease protein-coding gene in the regulatory network, suggesting that disease lncRNAs may contribute to the pathogenesis of disease by regulating some known disease genes. Their regulatory relations may enable us to discover novel disease lncRNAs using known disease genes.

## 5. Conclusion

In conclusion, we proposed a framework that integrates RNA-seq data and PPI network based on Bayesian network method to comprehensively characterize the functions of lncRNAs. By applying our method to RNA-seq data from prostate samples, we performed a large-scale functional prediction of lncRNAs and analyzed the features of regulatory relations between lncRNAs and protein-coding genes. Our study demonstrated that RNA-seq combining with PPI network based on Bayesian network method is a powerful method for functional analysis of poorly characterized ncRNAs and can be further used for mining functions of ncRNAs in other conditions.

## Supplementary Material

Supplemental Figures:
Figure S1. Differentially expressed lncRNAs and protein-coding genes in the regulatory network.
Figure S2. The overlapping functions between our approach and knockdown-based experiment for 38 lncRNAs.
Supplemental Tables:
Table S1. The numbers of reads, transcripts and genes in each RNA-seq sample.
