# Supplementary material for: Predicting the Functions of Long Noncoding RNAs Using RNA-Seq Based on Bayesian Network
Source: Biomed Res Int. 2015 Feb 28;2015:839590. doi: 10.1155/2015/839590 (PMC4359839; doi:10.1155/2015/839590)
Supplement: Supplementary file 1 — Supplemental Figures: Figure S1. Differentially expressed lncRNAs and protein-coding genes in the regulatory network. Figure S2. The overlapping functions between our approach and knockdown-based experiment for 38 lncRNAs. Supplemental Tables: Table S1. The numbers of reads, transcripts and genes in each RNA-seq sample. [file 839590.f1.doc]

**Supplemental figure**

**
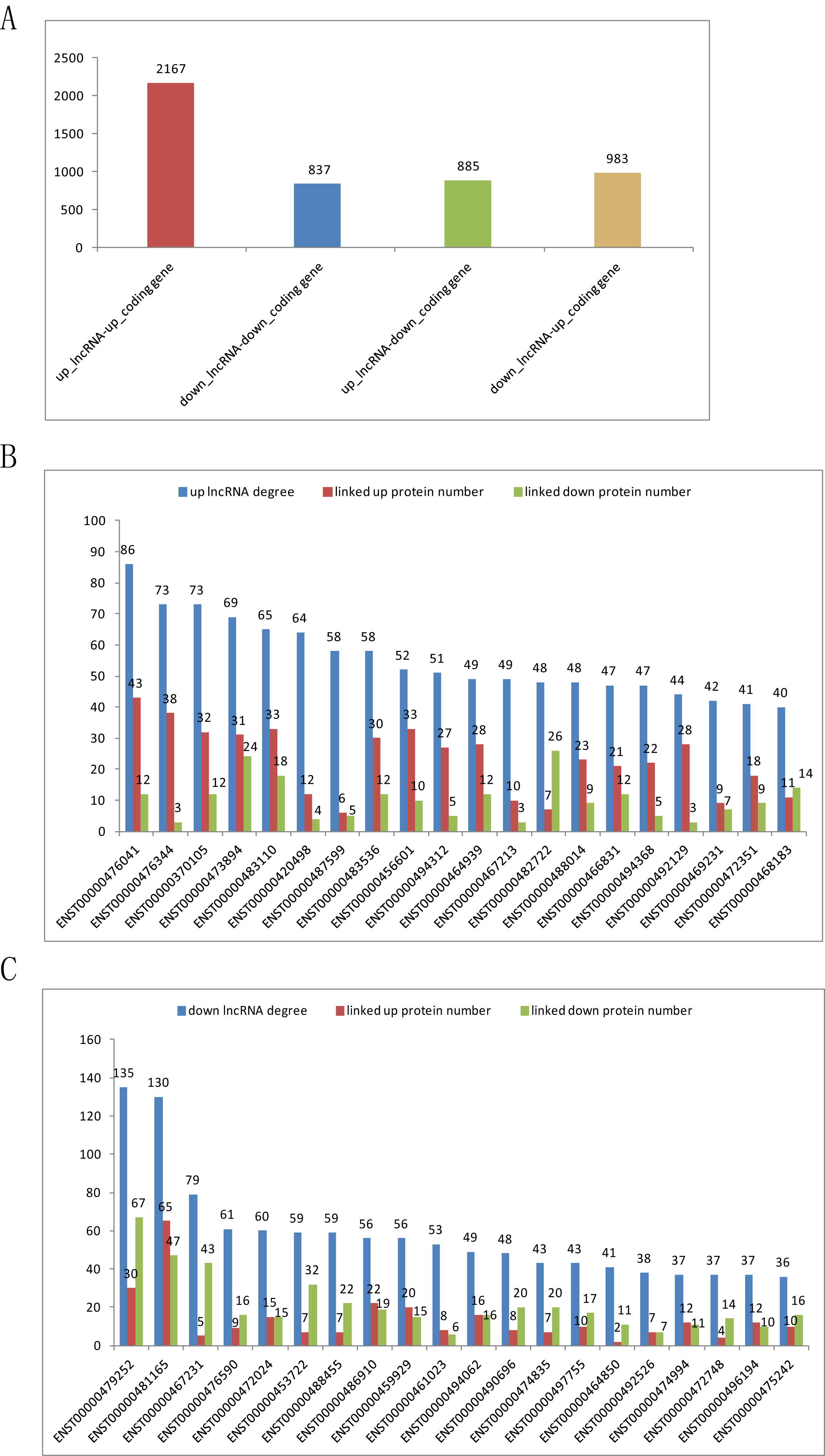
**

**Figure S1**. (A) The number of edges between differentially expressed lncRNAs and protein-coding genes in the regulatory network. (B) Degrees of the top 20 up-regulated lncRNAs with the numbers of their linked up-regulated and down-regulated protein-coding genes in the regulatory network. (C) Degrees of the top 20 down-regulated lncRNAs with the numbers of their linked up-regulated and down-regulated protein-coding genes in the regulatory network.


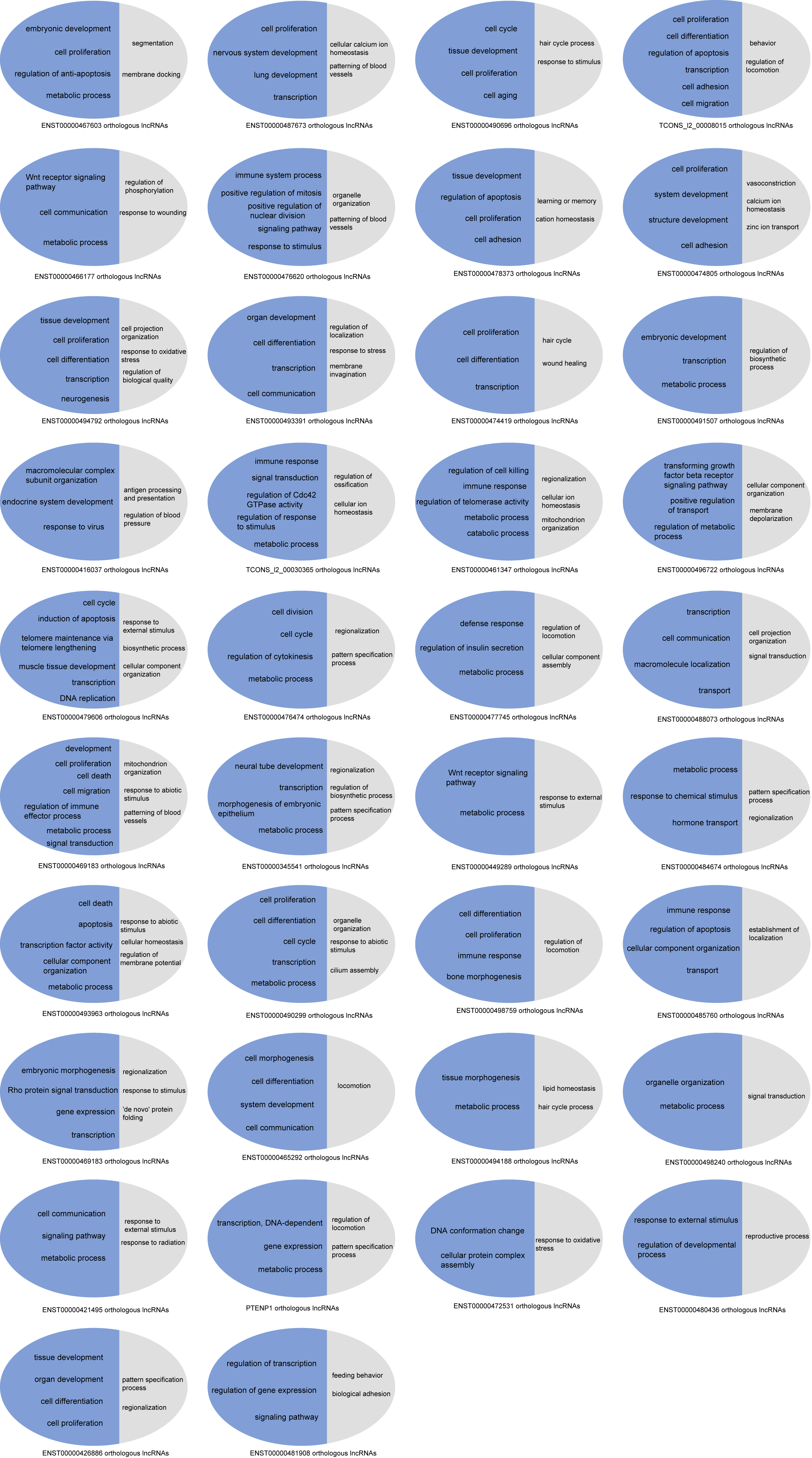


**Figure S2**. The overlapping functions between our approach and knockdown-based experiment for 38 lncRNAs. Blue color represents overlapping functions, and grey color represents functions only predicted by knockdown experiments.

**Supplemental Table S1: The numbers of reads, transcripts and genes in each RNA-seq sample.**

| **Sample Name** | **Reads** | **Transcripts** | **Genes** |
| --- | --- | --- | --- |
| **GSM618463_mctp_20F69AAXX_1** | 3014729 | 476097 | 28317 |
| **GSM618464_mctp_314NNAAXX_6** | 6732978 | 501862 | 29312 |
| **GSM618465_mctp_429T4AAXX_2** | 10304999 | 484692 | 27053 |
| **GSM618466_mctp_429T4AAXX_5** | 11450510 | 504288 | 27803 |
| **GSM618467_mctp_429T4AAXX_7** | 11463901 | 517875 | 31095 |
| **GSM618468_mctp_20BC5AAXX_8** | 3339064 | 468782 | 26187 |
| **GSM618469_mctp_20F69AAXX_2** | 3143829 | 468152 | 25988 |
| **GSM618470_mctp_30DJDAAXX_3** | 5966253 | 493077 | 28988 |
| **GSM618471_mctp_42TA8AAXX_7** | 10246940 | 521169 | 31465 |
| **GSM618472_mctp_42TA8AAXX_6** | 12349618 | 531406 | 34426 |
| **GSM618473_mctp_42TA8AAXX_5** | 11290288 | 526009 | 32416 |
| **GSM618474_mctp_42TA8AAXX_3** | 9735141 | 520401 | 31033 |
| **GSM618475_mctp_42TA8AAXX_2** | 10290767 | 521829 | 31519 |
| **GSM618476_mctp_42TA8AAXX_1** | 5695332 | 500101 | 28171 |
| **GSM618477_mctp_42TBDAAXX_8** | 7403279 | 510613 | 29196 |
| **GSM618478_mctp_429T4AAXX_3** | 11976502 | 513970 | 31375 |
| **GSM618479_mctp_20F0GAAXX_4** | 1940854 | 452416 | 24906 |
| **GSM618480_mctp_20F0GAAXX_1** | 1909339 | 451932 | 24817 |
| **GSM618481_mctp_20F0GAAXX_3** | 1935694 | 452979 | 24828 |
| **GSM618482_mctp_20F0GAAXX_2** | 1894327 | 452623 | 24767 |
| **GSM618483_mctp_20E6CAAXX_2** | 1870900 | 451362 | 24738 |
| **GSM618484_mctp_20E6CAAXX_3** | 2074057 | 453193 | 24952 |
| **GSM618485_mctp_20E6CAAXX_4** | 1741345 | 449495 | 24628 |
| **GSM618486_mctp_42PFAAAXX_6** | 9100281 | 507903 | 31209 |
| **GSM618487_mctp_42PFAAAXX_5** | 11147161 | 514375 | 31562 |
| **GSM618488_mctp_42PMUAAXX_6** | 8240819 | 505845 | 29885 |
| **GSM618489_mctp_42PMUAAXX_7** | 7471969 | 502980 | 29366 |
| **GSM618490_mctp_429T4AAXX_6** | 10975523 | 510976 | 30145 |
| **GSM618491_mctp_429T4AAXX_4** | 10519415 | 535634 | 35040 |
| **GSM618492_mctp_3064YAAXX_1** | 8135134 | 509342 | 29216 |
| **GSM618493_mctp_20F69AAXX_3** | 3323615 | 465170 | 25839 |
| **GSM618494_mctp_42B08AAXX_2** | 6266877 | 483963 | 25619 |
| **GSM618495_mctp_30CYNAAXX_5** | 1187704 | 437337 | 23913 |
| **GSM618496_mctp_209ENAAXX_8** | 1063070 | 435454 | 23698 |
| **GSM618497_mctp_314T1AAXX_1** | 5826261 | 494897 | 27286 |
| **GSM618498_mctp_30351AAXX_7** | 7228926 | 502193 | 28479 |
| **GSM618499_mctp_314T1AAXX_2** | 7219603 | 489406 | 26238 |
| **GSM618500_mctp_20E6CAAXX_6** | 2185721 | 461293 | 25327 |
| **GSM618501_mctp_20E6CAAXX_7** | 2214490 | 461689 | 25335 |
| **GSM618502_mctp_20E6CAAXX_8** | 1974122 | 459624 | 25047 |
| **GSM618503_mctp_20F0BAAXX_6** | 1995447 | 459131 | 25073 |
| **GSM618504_mctp_20F0BAAXX_7** | 2082374 | 459969 | 25160 |
| **GSM618505_mctp_20F0BAAXX_8** | 2055482 | 461426 | 25114 |
| **GSM618506_mctp_20F0GAAXX_7** | 2005245 | 460127 | 25042 |
| **GSM618507_mctp_20F0GAAXX_8** | 1982092 | 459690 | 25019 |
| **GSM618508_mctp_20F0GAAXX_6** | 2024605 | 459255 | 25036 |
| **GSM618509_mctp_30DJDAAXX_2** | 2100388 | 454443 | 25003 |
| **GSM618510_mctp_20CCAAAXX_7** | 1373958 | 442245 | 24296 |
| **GSM618511_mctp_20CCAAAXX_6** | 1333341 | 440839 | 24233 |
| **GSM618512_mctp_20CCAAAXX_4** | 1344809 | 441926 | 24248 |
| **GSM618513_mctp_20CCAAAXX_3** | 1384236 | 443647 | 24264 |
| **GSM618514_mctp_20CCAAAXX_2** | 1304722 | 441754 | 24205 |
| **GSM618515_mctp_20CCAAAXX_1** | 1093999 | 438676 | 23949 |
| **GSM618516_mctp_20E6CAAXX_1** | 1189170 | 440676 | 24107 |
| **GSM618517_mctp_20CCAAAXX_8** | 1287671 | 441191 | 24178 |
| **GSM618518_mctp_207D6AAXX_2** | 1103083 | 439564 | 24027 |
| **GSM618519_mctp_42B08AAXX_4** | 8600695 | 488867 | 26631 |
| **GSM618520_mctp_42B08AAXX_3** | 6300444 | 483376 | 25770 |
| **total** | 292413098 | 1692037 | 59404 |
